# Establishment of an intragastric surgical model using C57BL/6 mice to study the vaccine efficacy of OMV-based immunogens against *Helicobacter pylori*

**DOI:** 10.1242/bio.060282

**Published:** 2025-06-23

**Authors:** Sanjib Das, Prolay Halder, Soumalya Banerjee, Asish Kumar Mukhopadhyay, Shanta Dutta, Hemanta Koley

**Affiliations:** Division of Bacteriology, ICMR-National Institute of Cholera and Enteric Diseases, P-33, CIT Road, Scheme-XM, Beliaghata, Kolkata 700010, India

**Keywords:** Animal model, Surgical intervention, Gastric illness, Outer membrane vesicles, Vaccine efficacy, *Helicobacter pylori*

## Abstract

Chronic gastritis is one of the major symptoms of gastro-duodenal disorders typically induced by *Helicobacter pylori* (*H. pylori*)*.* To date, no suitable model is available to study pathophysiology and therapeutic measures accurately. Here, we present a successful surgical infection model of *H. pylori-*induced gastritis in C57BL/6 mice that resembles features of human infection. The proposed model does not require any preparatory treatment other than surgical intervention. C57BL/6 mice were injected with wild-type SS1 (Sydney strain 1, reference strain) directly into the stomach. Seven days post infection, infected animals showed alterations in cytokine responses along with inflammatory cell infiltration in the lamina propria, depicting a prominent inflammatory response due to infection. To understand the immunogenicity and protective efficacy, the mice were immunized with outer membrane vesicles (OMVs) isolated from an indigenous strain with putative virulence factors of *H. pylori* [A61C (1), *cag+/vacA s1m1*]*.* In contrast to the non-immunized cohort, the OMV-immunized cohort showed a gradual increase in serum immunoglobulin(s) levels on the 35th day after the first immunization. This conferred protective immunity against subsequent challenge with the reference strain (SS1). Direct inoculation of *H. pylori* into the stomach influenced infection in a short time and, more importantly, in a dose-dependent manner, indicating the usefulness of the developed model for pathophysiology, therapeutic and prophylactic studies.

## Teaser

**Summary:** This paper introduces an animal model using surgical intervention by directly injecting the inoculum to the stomach. This resulted in severe infection in 7 days. The model is efficient for prophylactic and therapeutic studies.

## INTRODUCTION

Gastroduodenal disorders are the cumulative effect of carefully orchestrated molecular interactions between host and pathogen factors belonging to the genus *Helicobacter* (Beswick et al., 2006). With almost 50% of the population worldwide infected by the pathogen, it is one of the major health burdens in developing nations (Salih, 2009). Although *Helicobacter pylori* has been recognized as a class I carcinogen by the WHO, very little has been explored thus far. This is primarily due to the asymptomatic nature of infected individuals, expensive clinical detection (e.g. endoscopy, urea breath test, etc.) and diagnosis with considerable information scarcity (Graham et al., 1991). In addition to this, the global antimicrobial resistance (AMR) pattern of *H. pylori* is changing alarmingly, resulting in a paradigm shift in ‘treatment of choice’ by clinical practitioners (Sukri et al., 2021).

Research in *in vitro* and *in vivo* systems of *H. pylori* is continuously enriching our understanding of pathophysiology and genetic predisposition related to adaptation, survival and coevolution of the pathogen (Abadi, 2017; Kusters et al., 2006). For instance, *H. pylori* has the inherent ability to modulate the gastric microenvironment, such as increasing gastric pH by means of urease upregulation, employing different adhesion proteins or simply dislodging itself when the pH becomes overwhelmingly acidic (Mobley, 2001; Bugaytsova et al., 2017). Such responses, along with others, act as the precursor to a chronic infection that largely depends on the gastric acid neutralizing capacity unique to each strain. The pathogen is known to recruit different adhesins depending upon the stages of disease progression, such as BabA during early infection or SabA during ongoing inflammation (Doohan et al., 2021). In addition, host antigens present on the surface of host cells, mucins and other gastric cells, such as A/B-Le^b^, MUC5AC, MUC1 and H type 1, play important roles in bacterial adhesion, further promoting the severity of different gastric maladies (Niv, 2015; Celli et al., 2009).

To date, a combination of antibiotics with a proton pump inhibitor (PPI) is the only mode of treatment available due to the lack of a potent vaccine (Sutton and Boag, 2019). Moreover, an efficient animal model is crucial to understanding the immunological attributes of different immunogen(s) for vaccine development, which existing models fail to satisfy. To date, considerable efforts have been made to establish a reliable murine (gerbil or mouse) model to serve this purpose, including extensive application of transgenic animals with single or double mutations, but unfortunately, no significant efforts have been made toward the route of administration to induce an infection (Toyoda et al., 2014). The preexisting method relies on the oral administration of multiple doses of inoculums along with antibiotic pretreatment to induce an infection (Dey et al., 2021). Moreover, it takes a minimum of 2 weeks to develop an infection in an animal model using the traditional approach, which is significantly higher than any other enteric pathogens, such as *E. coli* or *Salmonella* (He et al., 2021; Pfeifhofer-Obermair et al., 2022; Werawatganon, 2014).

Therefore, in this study, we introduced an infection by surgically exposing the stomach of C57BL/6 mice and directly injecting *H. pylori* inoculums. We assessed different pathological and immunological markers for active infection and applied the same to study the vaccine efficacy of outer membrane vesicle (OMV)-based immunogens isolated from a prevalent strain.

## RESULTS

### Characterization and selection of *H. pylori* strains used in the study

A total of 12 strains, including three reference strains and nine clinical strains, were checked for the presence or absence of major virulence genes i.e. cytotoxin-associated gene or *cagA* representing cag pathogenicity island (cagPAI), vacuolating toxin A or *vacA,* blood group antigen binding adhesin 2 or *babA2* and duodenal ulcer promoting gene or *dupA.* A type I or type II strain is defined by the presence of *cag* and allelic variations of *vacA* with signal region (*s1* or *s2*) and middle region (*m1* or *m2*)*.* A *cag+s1m1* is considered to be more virulent, thereby more strongly influencing disease development than *cag+s2m2* or any combination of *s1, s2, m1* and *m2*. Additionally, allelic variations of *babA*, i.e. *babA2*, plays a key role in adhesion to the Lewis B (Le^b^) antigen of blood as *babA1* is known to be non-functional (Ghosh et al., 2016). *dupA* belongs to a plasticity region (*jhp0917-jhp0918*) and has been found to be responsible in developing ulcers in *H. pylori* infected individuals (Lu et al., 2005). Therefore, any strain positive for all these genes can be considered to be more virulent than others. A61C (1) is positive for all these virulence genes and therefore is selected for immunogen preparation. However, for model establishment and challenge study purpose, SS1 is considered to be more suitable than others as it is a mouse adapted strain. The result of genetic characterizations of all strains is listed in a table (Table S3).

### Clinical response caused by surgical intervention

In the present study, 2×10^8^ CFU of bacteria were used to induce an active infection. Oral inoculation with this dose revealed inconsistent results. Moreover, in the majority of cases, very little or no recovery of the bacterial population was observed using available detection techniques. Mice receiving WTSS1 directly to their stomachs by surgical means developed various degrees of gastric changes. Recovery of bacterial colonies from stool was insignificant and erroneous compared to gastric tissues, which were considerably higher (∼2–3 times) and were confirmed to be positive upon rapid urease test (RUT), spread-plate and polymerase chain reaction (PCR). The recovery rate of *H. pylori* from the 7-day-infected mice was comparatively higher than that at 14 days post infection (Fig. 2).

**Fig. 1.**
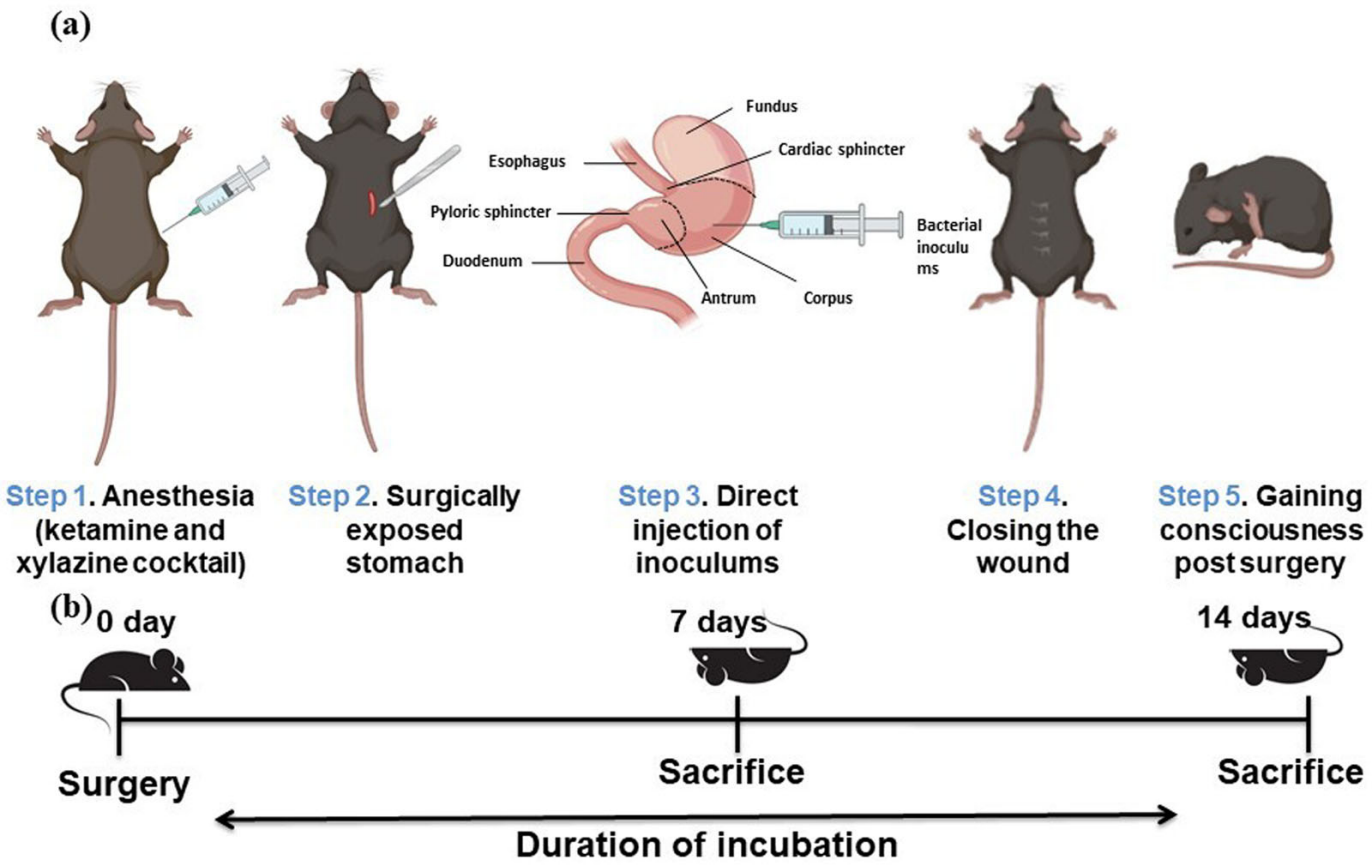
**Schematic diagram of the intragastric infection model in C57BL/6 mice.** (A) Graphical representation of the ‘surgical model’ using C57BL/6.Bacterial inoculation (∼2×10^8^CFU/ml) is directly injected into the stomach. (B) Schematic schedule from infection to euthanasia.

**Fig. 2.**
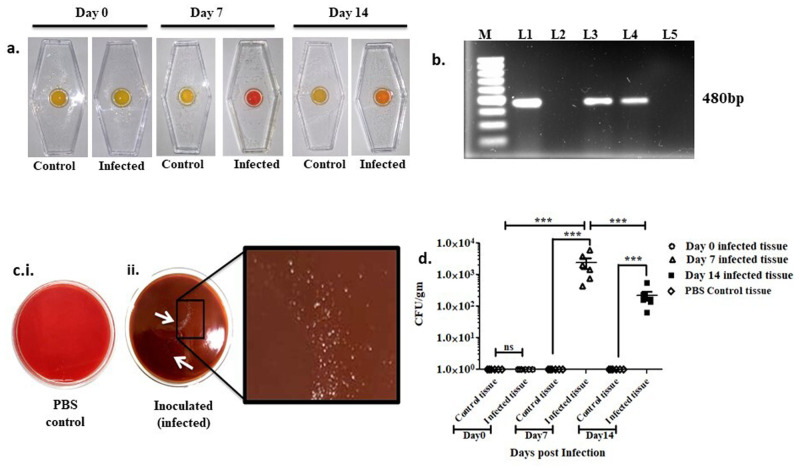
**Intragastric infection induced by wild-type (WT) SS1 observed through urease test, *ureB* PCR, colonization in gastric tissue.** (A) RUT of infected gastric tissue; day 0, day 7 and day 14 with respective PBS control. (B) Confirmatory *ureB* PCR for the presence of *H. pylori* recovered from the gastric tissue of infected mice. M-100 bp marker, L1-positive control, L2-day 0 post infection, L3-day7 post infection, L4-14-day post infection, L5-PBS control. (C) Blood agar plates showing (i) plate containing no *H. pylori* colonies recovered from gastric tissue of 7 days post PBS inoculated mice (ii) plate containing *H. pylori* colonies recovered from gastric tissue of 7 days post SS1 infected mice. (D) Colonies recovered from mice of 0, 7- and 14-days post infection. Data represented here are the mean values +/-standard deviation (SD) of three independent experiments. The differences in day-wise response of each colonization assay were highly significant with respect to PBS control tissue. Statistical significance was found between 0-day, 7-day and 14-day infected mice tissue (***P<0.001; ns, non-significant).

### Intragastric surgical evoked inflammatory response

**Fig. 3.**
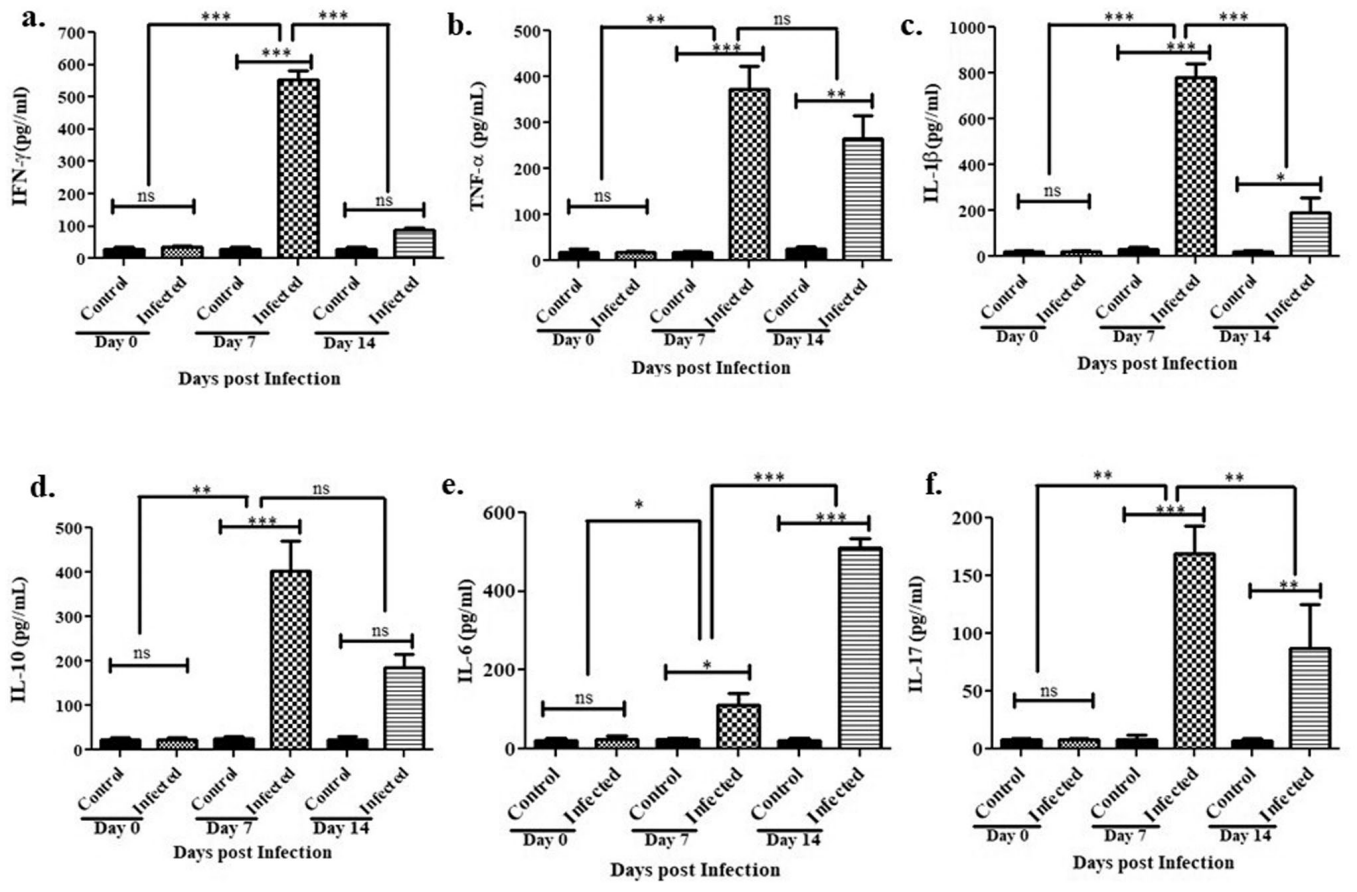
***Helicobacter pylori;* WT SS1 induces the production of cell mediated cytokines responses post-surgical intervention.** (a) IFN-γ, (b) TNF-α, (c) IL-1β, (d) IL-10, (e) IL-6, (f) IL-17 cytokines in serum isolated on day 0, day 7 and day 14 post-surgical infection with respective PBS controls. All cytokines are measured by ELISA (*n*=6). Statistical analyses were performed using the non-parametric Student's *t*-test (Mann–Whitney tests) to evaluate data; (***P-value<0.001; **P-value<0.01; *P-value<0.05; ns, non-significant), each bar represents median and error values of six±s.e. of three independent experiments.

Cytokine analysis of intragastrically infected mice of different time points i.e. 0 days, 7 days, and 14 days, showed drastic differences in serum cytokine levels. IFN-γ, IL-1β, TNF-α, IL-10, and IL-17 are increasing more on day 7 after infection and also reducing progressively on day 14. In case of IL-6, which is responsible for sustaining inflammation is increasing on day 14. The majority of the pro-inflammatory cytokines were upregulated after 7 days post infection, except IL-6, which was found to be more pronounced at 14 days than at 7 days post infection (Fig. 3) indicating active *H. pylori* infection.

### Histopathological changes due to intragastric infection

**Fig. 4.**
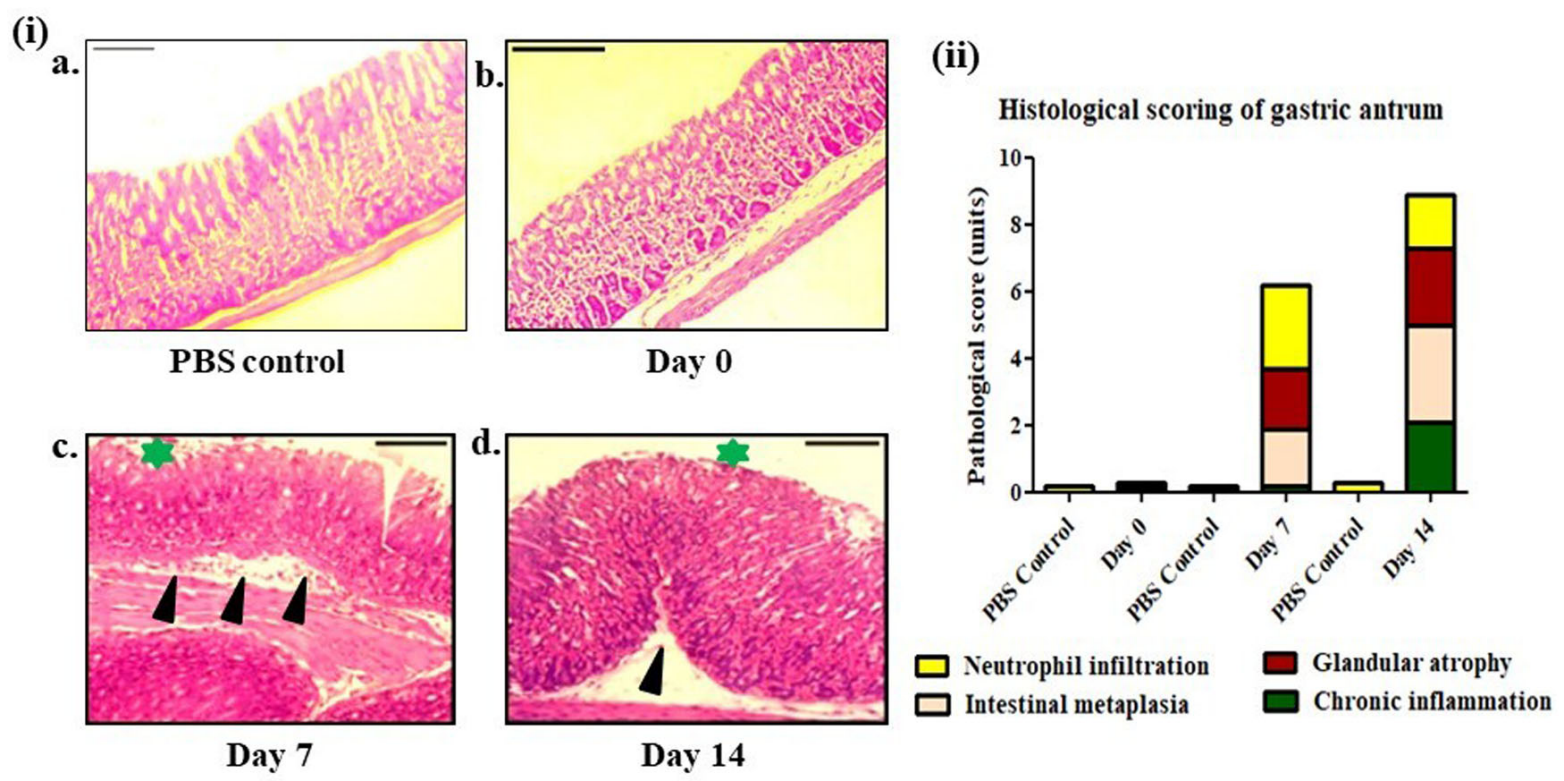
**Histological (H&E staining) observation and histological scoring of gastric epithelium after intragastric surgical infection with *H. pylori* (SS1).** (ia-d) All panels show the antrum part of the stomachs harvested from C57BL/6 mice. (ia) no distinct changes observed in PBS control, mice receiving PBS only, (ib) zero inflammation in day 0 post-surgical infection of C57BL/6 mice, (ic) severe inflammatory cell infiltration, glandular atrophy, intestinal metaplasia of 7 days post-surgical infection of C57BL/6 mice, (id) mild to moderate inflammatory cell infiltration, disruption in epithelial lining, glandular atrophy, chronic inflammation in 14 days post-surgical infection of C57BL/6 mice. Images were captured at 20× magnification. Scale bars: 100 μm. Inflammatory cell infiltration indicated by (black arrowhead), gastric epithelial damage (green star). (ii) Histopathological scoring is done according to Huston updated Sydney classification system. Colored histogram represents the mean scores of histological scoring of experimental animals (*n*=6) and PBS controls (*n*=6). All experiments were performed in triplicate.

Histopathological observation plays crucial role in *H. pylori* diagnosis. *H. pylori* infection causes local inflammation in gastric tissue marked by various degrees of inflammatory infiltration with substantial damage in gastric epithelium leading to the survival of the bacteria to the gastric microenvironment. Moreover, previous study has already showed a pronounced effect on gastric tissue of C57BL/6 mice upon *H. pylori* infection. Therefore, to establish a successful infection mediated by surgical intervention, stomach samples were taken at different time points and the topographical changes were compared. Negative control mice, receiving only PBS and day-0 mice had no inflammation (Fig. 4ia,b), whereas inflammatory infiltration was significantly higher on day 7 (Fig. 4ic) than on day 14 (Fig. 4id). In addition to this, mucosal epithelium was severely damaged with exposed gastric pits in both cases. In contrast, metaplasia due to infection was more prominent on day 14 than day 7, indicating a successful infection. Histopathological scoring was assigned based on Sydney system (Fig. 4ii).

### Isolation and characterization of OMVs from *H. pylori* strain A61C (1)

**Fig. 5.**
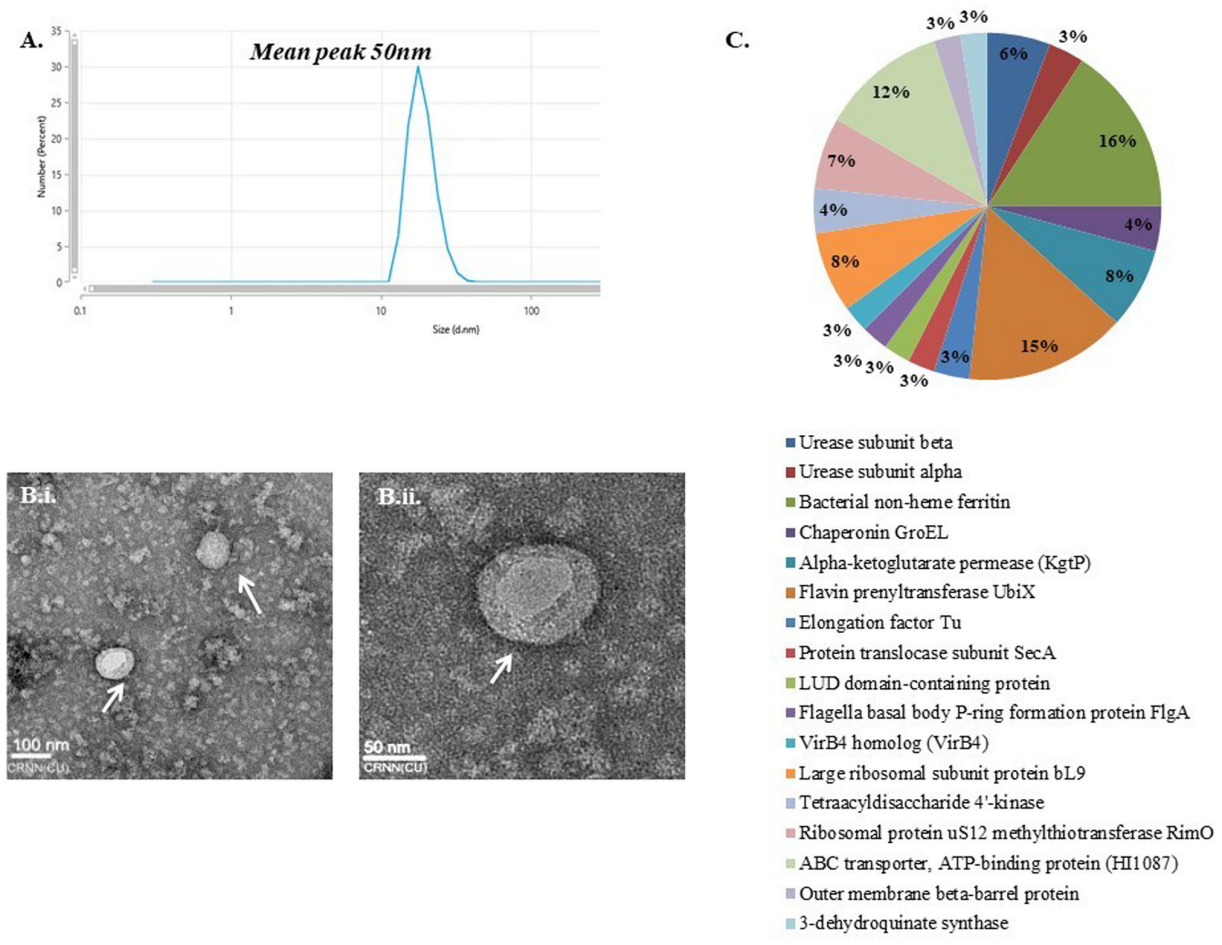
**Characterization of *H. pylori* OMVs isolated from strain A61C (1).** (A) Dynamic light scattering showing a uniformity in OMVs population with mean peak at 50 nm. (B) TEM images revealing the circular morphology of *H. pylori* OMVs of A61C (1) strain. (Bi) Image taken at 100 nm scale. (Bii) Image taken at 50 nm scale. Both TEM images revealed the thick bilayer structure with hollow center of the OMVs. (C) Percentage of major proteins present on OMVs.

The OMVs isolated from the broth culture of A61C (1) were purified and assessed using dynamic light scattering (DLS), transmission electron microscopy (TEM) and proteomics analyses using LC/MS (Fig. 5A). The data revealed uniformity in OMVs structure with a diameter of 50 nm (Fig. 5Bi, ii). TEM image showed the OMVs to be circular in shape with distinct bilayers. The protein components present in OMVs isolated from the immunogen strain [A61C (1)] revealed 18 major proteins including UreB, UreA, FtnA, GroEL, UbiX, Tuf, SecA, RplI, LpxK, RimO, AroB along with some other proteins with unknown localization (Fig. 5C; Table S4). The presence of proteins like UreA, UreB and GroEL on OMVs indicates the potential to generate a strong immune response as these proteins are known for their immunomodulatory activities. The sub-cellular localization of proteins indicated by the software includes cytoplasm, membrane, periplasmic space and plasma membrane of the bacteria. *H. pylori* LPS is known to have no cytotoxicity, which is also evident from the cytotoxicity assay we performed (Fig. S2).

### *H. pylori* OMVs induce pro-inflammatory cytokine response

**Fig. 6.**
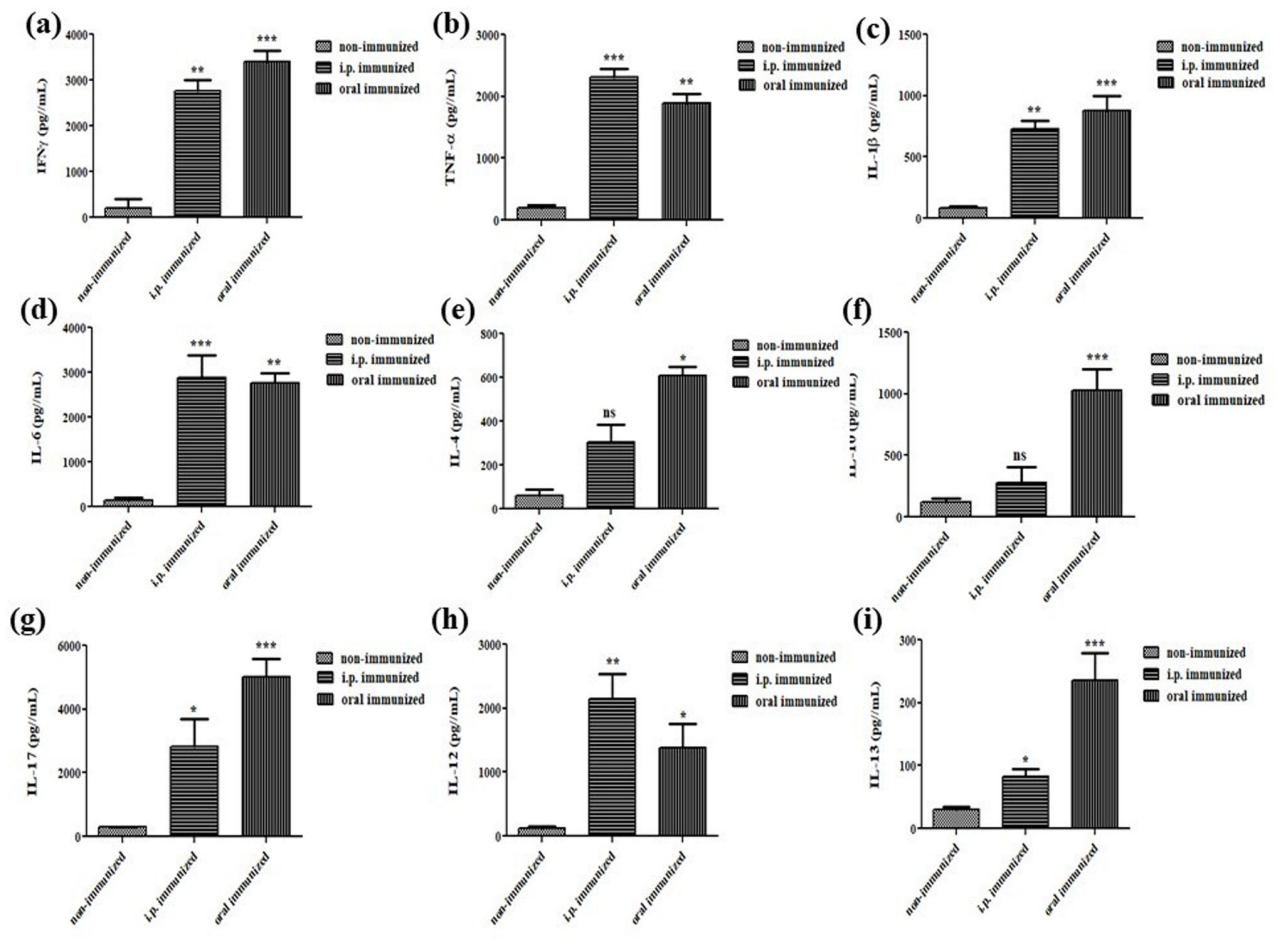
***H. pylori* OMVs induces the production of cell mediated cytokines responses.** (A) IFN-γ, (B) TNF-α, (C) IL-1β, (D) IL-6, (E) IL-4, (F) IL-10, (G) IL-17, (H) IL-12, (I) IL-13, cytokines in culture supernatant of *ex-vivo* cultured splenic cells of immunized and non-immunized (PBS immunized) mice after 24 h of re-stimulation with OMVs. The differences in immunized (intraperitoneal and oral immunization) mice serum response of each of the studied cytokines were highly significant than non-immunized. All cytokines are measured by ELISA (*n*=6). Statistical analyses were performed using the non-parametric Student's *t*-test (Mann–Whitney tests) to evaluate data; (***P-value<0.001; **P-value<0.01; *P-value<0.05; ns, non-significant). Each bar represents median and error values of six±s.e. of three independent experiments.

Thirty-fifth day post-immunization splenic cells were harvested from both immunized and non-immunized mice and re-stimulated with 50 μg of OMVs. A significant induction in IFN-γ, TNF-α, IL-1β, IL-4, IL-10, IL-17, IL-6 and IL-12, IL-13 levels were observed (Fig. 6a–i). Moreover, our data revealed oral immunization to be a better route for immunization as most of pro-inflammatory cytokines. Contrary to previous studies (Liu et al., 2019), our study did not find a Th1- or Th2-biased response indicating the immune response against OMVs is independent of routes of administration, compared to the control, both orally and intraperitoneally immunized animals revealed elevation in cytokines. Altogether, immunization invoked a wider array of cytokines than non-immunization, implying the potential of OMVs as a vaccine candidate.

### Immunization of *H. pylori* OMVs elicited higher adaptive immune response

**Fig. 7.**
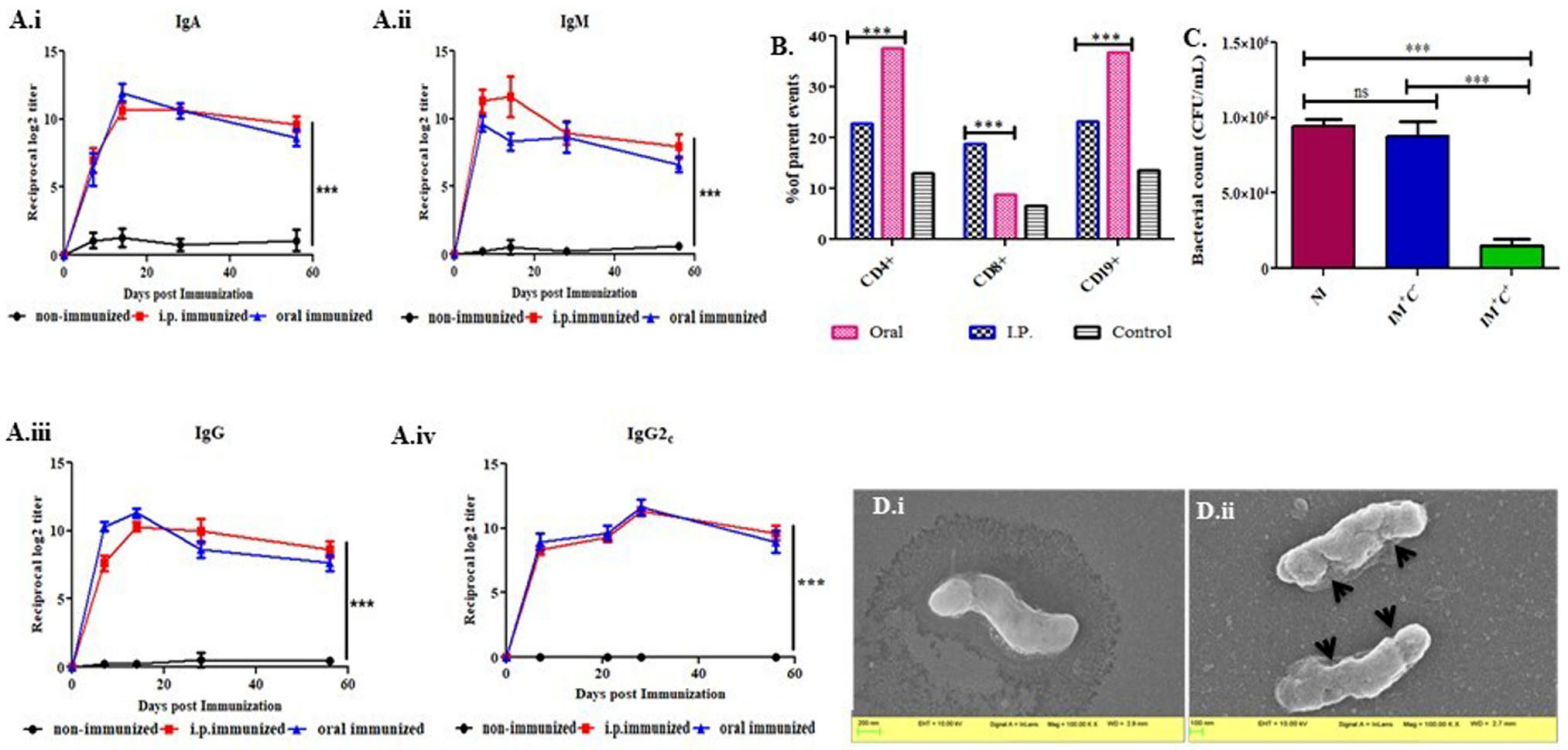
**Reciprocal log2 titer of serum IgA, serum IgM, serum IgG, and serum IgG2c immunoglobulins from *H. pylori* OMVs immunized and non-immunized (PBS immunized) group against OMPs.** Immunization induces the population of CD4+, CD8+, and CD19+ splenic cells of immunized over non-immunized (PBS immunized) mice and the microscopic image of serum bactericidal activity of immunized and non-immunized. Mouse serum IgA (Ai), serum IgM (Aii), serum IgG (Aiii) and serum IgG2c (Aiv) were measured separately after three doses of intraperitoneal or oral immunization against OMP of *H. pylori.* (B) Bar diagram represents the percentage of CD 19+, CD 4+, and CD 8+ spleen cells from immunized and non-immunized mice using FACS analyses. Significant statistical difference was found between OMVs immunized and non-immunized spleen cell population (***P-value<0.001). (C) OMVs immunized mouse serum is effective in complement mediated lysis of *H. pylori* (SS1). *H. pylori* (SS1) was separately incubated with OMVs immunized serum or non-immunized serum with or without guinea pig complement for 1 h at 37°C. Viable bacterial count was determined by spread-plate method. Two-way ANOVA test was used for statistical analysis. Bars represent mean±s.e. of three individual experiments. (***P-value<0.001; ns, non-significant.). NI, non-immunized serum; IM+C−, OMVs immunized serum without complement; IM+C+, OMVs immunized serum with complement. (D) SEM images after serum bactericidal assay using non-immunized serum with complement (i) and immunized serum with complement (ii) (black arrowheads indicate immunized antibody-mediated lysis in presence of complement).

Previous studies on bacterial extracellular vesicles revealed OMVs to be an excellent vaccine candidate against bacterial pathogens (Song et al., 2020). OMVs are known to induce both humoral and cellular arms of immune responses usually mediated by outer membrane proteins (OMPs) and lipopolysaccharides (LPS). We investigated serum immunoglobulin levels (Fig. 7Ai–iv) of orally and intraperitoneally immunized mice and found significant difference from control. However, we did not find significant differences between oral and intraperitoneal immunization.

Next, we evaluated the bactericidal activity of the immunized serum. The data showed significant reduction in bacterial number when immunized serum is incubated with 25% guinea pig serum as compared to non-immunized mice serum (Fig. S3). This confers activation of complement mediated pathway, along with sufficient antibody titer in immunized C57BL/6 mice that effectively kill the bacteria by damaging the bacterial surface as viewed under SEM (Fig. 7C, Di, ii). Comparative analyses of the splenic cell population of immunized and non-immunized mice were done using a flow cytometer. Immunization with OMVs showed significantly higher population of CD4+, CD8a+ and CD19+ (Fig. 7B) cells indicating a strong immune response in immunized mice.

In all, immunization with *H. pylori* OMVs generated adaptive immune responses in C57BL/6 mice and significantly activated adaptive immune responses, which could in turn help to provide a long-term protective immune response against infections caused by *H. pylori* (Fig. 7Aiii, iv).

### Protective efficacy study post immunization

**Fig. 8.**
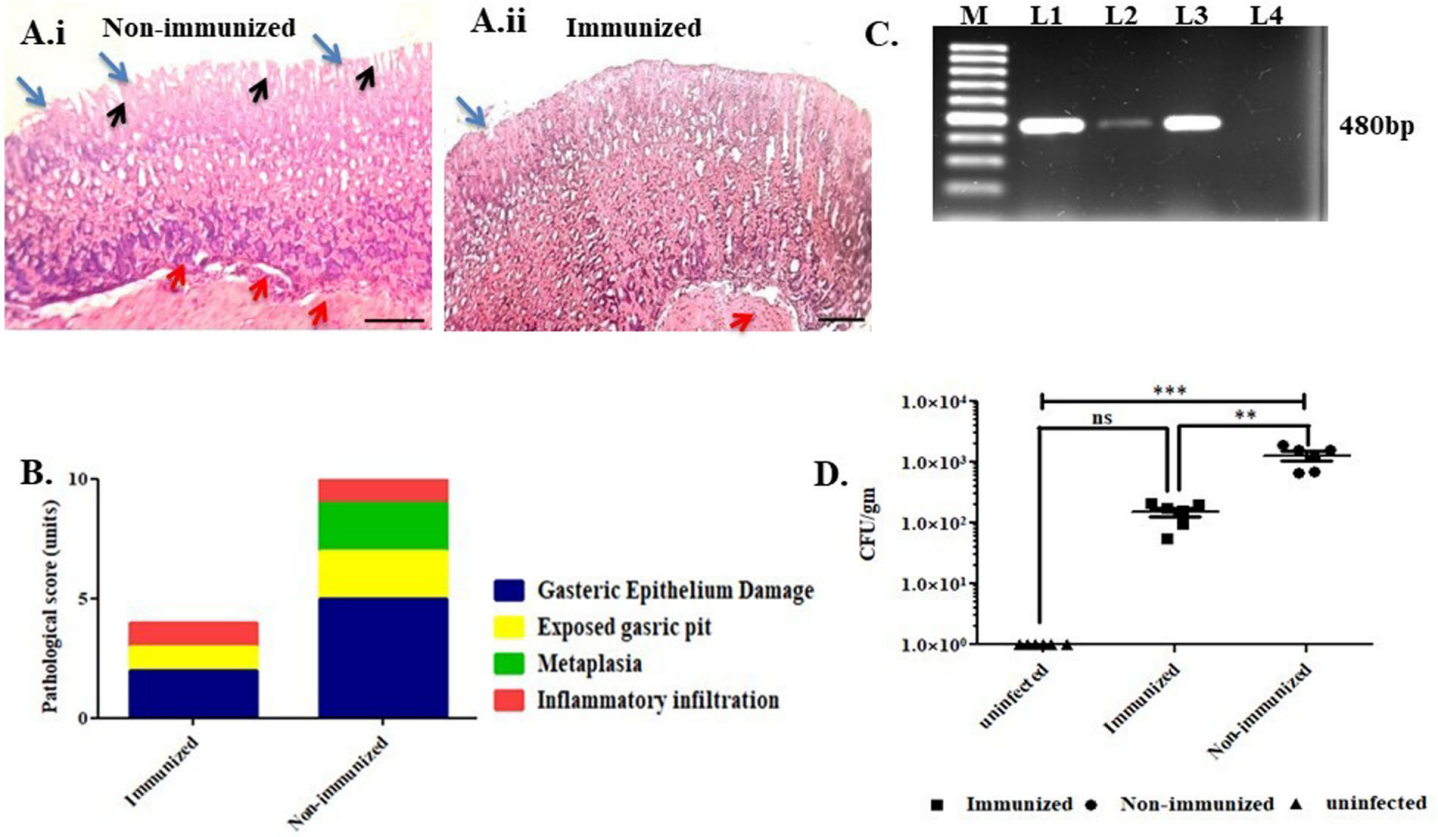
**OMVs immunized mice shows reduced gastric tissue damage, inflammation after infection with SS1 (2×10^8^ CFU) and reduce bacterial colonization in gastric tissue.** Histological images represent both (Ai) non-immunized (PBS immunized) antrum of stomach and (Aii) immunized antrum of stomachs. OMVs immunized mice showed mild epithelial layer damage, less altered gastric mucosa and inflammatory infiltration, whereas non-immunized mice displayed marked epithelial damage, inflammatory infiltration, exposed gastric pit and early signs of gastric metaplasia. Blue arrow, gastric epithelium; black arrow, exposed gastric pit; red arrow, inflammatory infiltration. (B) Pathological scores of immunized or non-immunized mice post *H. pylori* SS1 challenge. (C) *ureB* PCR shows significant changes in bacterial DNA yield harvested from gastric tissues of both non-immunized and immunized mice post intragastric surgical challenge; M, 100 bp ladder; L1, positive control; L2, immunized mice gastric tissue; L3, non-immunized mice gastric tissue; L4, negative control. (D) *H. pylori* colonization in gastric tissue of immunized and non-immunized mice 7 days post challenge.

After immunization with OMVs, the immunized and non-immunized animals were challenged with WT SS1 (2×10^8^ CFU), and the colonization was analyzed 7 days post infection. Significantly less colonization was observed in the stomach tissue of immunized than non-immunized animals indicating a substantial reduction in bacterial load (Fig. 8D). To confirm this, DNA was extracted from the gastric tissue and subjected to *ureB* PCR for the presence of *H. pylori.* All non-immunized mice were found to be positive, whereas in immunized animals no significant presence of *H. pylori* DNA was found (Fig. 8C). Histopathological changes of both immunized and non-immunized mice stomachs were analyzed 7 days post infection using surgical intervention (Fig. 8Ai, ii). OMVs immunized mice showed a significant reduction in gastric epithelial damage, altered gastric mucosa, inflammatory infiltration, exposed gastric pit, and metaplasia. Pathological scores were also less in immunized mice than non-immunized mice (Fig. 8B). Overall, reductions in bacterial numbers were observed upon immunization.

## DISCUSSION

Over the years, a number of animal models have been evaluated for pathophysiology or treatment against *H. pylori*, including gnotobiotic pigs, dogs, cats, Mongolian gerbils, guinea pigs, rhesus monkeys, and mice (Eaton et al., 1998; Krakowka et al., 1998; Neiger and Simpson, 2000; Watanabe et al., 1998; Wu et al., 2008; Sturegård et al., 1998; Shomer et al., 1998; Solnick et al., 2003, 2006; Asgari et al., 2022; Panthel et al., 2003; Pan et al., 2018). In most cases, C57BL/6 or black mice were explored extensively because of their substantial contribution in *H. pylori-*related studies. The proper route for administering the pathogen and/or immunization also has a key impact on the development of an infection and assessment of immune response, which is another important consideration when selecting an animal model for *in vivo* studies. Therefore, cytokine alterations along with histological changes described in the present study represent a successful infection achieved through our newly developed surgical model. Conventional approaches for studying *H. pylori* infection in animals usually involve multiple oral inoculations using an oral gavage (Nedrud, 1999; Werawatganon, 2014; Taylor and Fox, 2012). However, relying solely on the oral route to induce an infection and expecting the bacterium to outcompete the existing microflora and successfully colonize the stomach may not always yield a consistent result in any given experimental setting. This can require significant time and resources and nonetheless foster uncertainty about the actual infective status of experimental animals. Therefore, it is important to consider the limitations and variability of the *in vivo* systems and look for alternative approaches that could provide a more reliable method of establishing *H. pylori*-mediated pathogenesis in animal models. Clinical detection of *H. pylori* infection generally involves histology and PCR apart from the RUT (Guo and Mekalanos, 2002; Mobley, 2001; Lee et al., 2015). Serological tests are often avoided, as previously invoked antibodies fail to recognize the actual infective status of recent manifestations (Lindsetmo et al., 2008; Raj et al., 2017). As a consequence, this increases the chances of false positive results. In addition, histology allows visualization of pathogen-induced changes in gastric tissues, such as the intensity of inflammatory cell infiltration or aberrations in gastric topology, while PCR detects the presence of genomic DNA of *H. pylori* in gastric tissue samples (Jara et al., 2022). However, it should be noted that neither histological observations nor negative PCR results rule out the presence of an infection (Taylor and Fox, 2012). Thus, a number of different techniques must be employed simultaneously to achieve a more accurate diagnosis of *H. pylori* infection (Wang et al., 2015). Our study comprised a combination of histological observation, PCR detection and quantification of serum cytokine levels to confirm active *H. pylori* infection.

Surgical intervention initially spiked pro-inflammatory cytokines such as IFN-γ and IL-1β along with IL-17 significantly more on day 7 than on day 14. However, as the infection progressed, these cytokines were lowered and finally balanced, except for IL-6, which was found to be elevated more on day 14 than on day 7. A pronounced IL-6 level at later stages might indicate ongoing inflammation in the gastric lining with potentially developing chronic gastritis. Such responses were further supported by the induction of other cytokines. IFN-γ is an early effector molecule responsible for generating a Th1-mediated response by initiating different signaling cascades. However, upregulation of IFN-γ transiently downregulates IL-1β production. In addition, IL-17, a cytokine regulating the Th-17-based response, plays important roles in both pathogenesis and host immunity. Studies with chronic diseases have revealed that well-balanced IL-1β and IL-17 levels are constitutively produced to sustain inflammation due to infection in the long term (Coccia et al. 2021). In the case of *H. pylori* infection, both IL-1β and IL-17 play crucial roles in pathogenesis; in particular, IL-17 influences the disease outcome upon infection. Our model showed an initial elevation in these cytokines, which decreased over time, indicating progression toward a chronic infection. However, as 7 days were not sufficient to develop a chronic infection, our model showed promising results in a time-dependent manner. Consistent with the cytokine analysis, histopathological observations also validate such changes to some extent. Intense inflammatory cell infiltration was observed on day 7 than on day 14, and the gastric lining was found to be more damaged with exposed gastric pits, indicating destruction caused by bacteria. Nevertheless, we did not find any striking structural abnormalities in gastric tissue 7 days post infection. PCR results from the same samples confirmed the presence of bacterial genomic DNA in experimental animals.

Next, we evaluated the surgical model for vaccine efficacy studies. Two different immunization routes were assessed to observe any alterations in the immune response due to changes in the route of administration. Immunization was performed both orally and intraperitoneally on days 0, 14 and 28. Initially, an elevation of serum IgG, IgM and IgA levels was observed against OMPs but not LPS of *H. pylori*. This can be due to the structural similarity between *H. pylori* LPS and blood antigens of the host (Yokota et al., 2007; Muotiala et al., 1992). Furthermore, we evaluated IgG2c (IgG subtype) and found it to be increased in immunized rather than non-immunized groups (Song et al., 2020). Our study found oral immunization to be better responsive than the intraperitoneal route, which can be due to the presence of different surface proteins on OMVs that are more readily absorbed and reactive to gastric epithelial cells than peritoneal immune cells. A splenic cell re-stimulation (*ex vivo*) assay revealed enhanced Th2-based cytokine responses, such as IL-4, IL-13, IL-10 and IL-12, coinciding with previous studies with *H. pylori*-derived OMVs used as immunogens (Liu et al., 2019). Interestingly, our study did not find any biased immune response against OMVs, indicating that the immune response to OMVs is not general but rather unique to each strain. CD4+, CD8a+ and CD19+ cell populations were increased due to OMV immunization independent of the route of administration. OMV immunization ultimately leads to a reduction in bacterial colonization in immunized animals but not in non-immunized animals. Serum bactericidal assay (SBA) typically denotes the functional aspect of immunogen-invoked antibody response in killing the bacterial population via complement mediated pathway. This *in-vitro* method involves incubation of bacteria in presence of heat-inactivated serum isolated from both OMVs immunized and PBS immunized mice. Antibodies generated in hosts due to immunization are sufficient to reduce the bacterial CFU by means of agglutination as demonstrated in OMVs induced immune response against *S. Typhi* and *Paratyphi A* (Howlader et al., 2018). However, agglutination does not directly imply a bacteriostatic or bactericidal activity of the antibodies. Therefore, purified baby rabbit (Halder et al., 2023) or guinea-pig (Banerjee et al., 2023) antibodies were added to complement these antibodies and ensure the lysis of the bacteria via complement-mediated pathway. In the present study, incubation of bacteria treated with OMVs immunized or PBS immunized sera in presence of guinea-pig complement lead to significant reduction in viable colony numbers in immunized compared to the PBS immunized mice groups.

In conclusion, the intragastric surgical model of *H. pylori* infection can be used to study the pathophysiology, immune response, and potential therapies for *H. pylori* infection. Our study indicates that a minimum of 7 days is enough to develop an infection in this model. All experimental results showed that tissue samples collected at 7 days post infection can provide better results for diagnosing *H. pylori* infection than samples obtained at 14 days post infection, as histological changes and inflammatory cell infiltration are typically more pronounced at earlier time points post infection. Moreover, the cytokine response and antibody generation further support this model for vaccine efficacy studies. The immunization of mice with *H. pylori* OMVs has been shown to reduce the bacterial load with elevated antibody titers and protect gastric tissue from destruction. Therefore, the intragastric surgical model can become a valuable tool for understanding the pathophysiology of *H. pylori* infection, formulation and evaluation of potent vaccine candidates and development of potential therapeutics.

## MATERIALS AND METHODS

### Bacterial strains and culture conditions

Bacterial strains were revived from glycerol stock using brain heart infusion agar (BD Difco, USA) supplemented with 7% horse blood, 0.4% IsoVitaleX with antibiotics such as amphotericin B, trimethoprim, and vancomycin (Sigma-Aldrich, USA) at concentrations as described previously (Patra et al., 2012). Inoculated plates were then kept under microaerophilic conditions (5% O_2_, 10% CO_2_, and 85% N_2_ at 37°C) for 48 h and sub-cultured before conducting any experiment.

Broth culture was prepared using Brucella Broth (BD, Difco, USA) supplemented with 10% horse serum and vancomycin (Sigma-Aldrich, USA). The inoculated flask was then kept in shaking conditions (100 rpm) overnight while maintaining the microaerophilic environment (Whitmire and Merrell, 2012).

### Characterization and selection of strains

All strains were checked for oxidase, catalase and urease as mentioned elsewhere (Chattopadhyay et al., 2004). Next, an antibiogram was performed using the agar dilution method following CLSI guidelines (Table S1). PCR-based detection was applied for genotypic characterization. Some major virulence factors, such as *cagA*, *vacA*, *babA* and *dupA*, were checked using either simplex or multiplex PCR (Ghosh et al., 2016; El-Sayed et al., 2020). The primers used in the present study are listed in a Table S2.

### Animals

The NICED-Animal house facility supplied 6- to 8-week-old female C57BL/6 mice. The animals were maintained at 25±2°C with 65±2% humidity and a 12-h:12-h light:dark cycle. Animals weighing ∼22 g were selected for the study and provided with sterile food and water *ad libitum*. All experiments were performed following the standard operating procedure outlined by the Committee for the Purpose of Control and Supervision of Experiments on Animals (CPCSEA), Ministry of Environment and Forest, Government of India (CPCSEA registration number 68/GO/ReBi/S/1999/CPCSEA valid 17.07.2024) and Institutional Animal Ethics Committee (IAEC) of NICED was approved (approval number PRO/194/June 2022-25) and supervised experimental design and protocols from time to time.

### Animal experimental design

Thirty-six C57BL/6 mice were randomly assigned into two major groups, each comprising 18 animals. To determine an infectious dose for the surgical model, the first set of mice was further divided into three subgroups and infected with a dose of either 1×10^8^ (*n*=6) or 2×10^8^ CFU/mL (*n*=6, each) along with PBS control (*n*=6). All groups were housed for 1 or 2 weeks under sterile conditions.

For immunological studies, the remaining 18 mice were separated into two groups as: non-immunized (NI) (*n*=6) and orally or intraperitoneally immunized (IM) (*n*=6 in each group). An oral or intraperitoneal immunization with 50 μg of OMVs dissolved in PBS was administered on days 0, 14, and 28. Blood was collected at different time points, and the serum was isolated and stored at −20°C for use in different immunological assays. For the protective efficacy study, both groups (IM and NI) were infected surgically on the 35th day post-first-immunization and euthanized 7 days post-infection (Fig. S1).

### Intragastric surgical model development

Experimental animals were kept in fasting conditions overnight with sterile water. Initially, animals were sedated by an intraperitoneal injection of a mixture of ketamine (87.5 mg/kg) and xylazine (12.5 mg/kg) (IACUC, 2023). The stomach was exposed through a 2–3 cm midline incision without compromising any major blood supply. A disposable syringe with a 26G needle containing 200 μl (∼2×10^8^ CFU) of the inoculums in PBS was directly injected into the stomach. Hydration was maintained in the exposed stomach using sterile normal saline throughout the surgery. The stomach was placed back inside the abdominal cavity, and the incision was sutured back. The incision site was monitored for any infection and occasionally washed with 5% povidone-iodine (betadine) soaked in a sterile gauge for 72 h (Barman et al., 2011). Sterile food and water were provided to the animals once they regained consciousness (Fig. 1).

### Post-surgery observation

All infected mice were observed twice a day for 7 days. Physical parameters were checked along with stool consistency and the nature of mucus or blood (if any) present in the feces. Rectal swabs were taken daily and were subjected to RUT solution and spread-plate to observe the shedding of the organism. Isolated colonies (if any) were confirmed using a PCR-based technique. *H. pylori* infection augments the modulation of both pro- and anti-inflammatory cytokines in the host (Jan et al., 2021). Therefore, IL-1β, TNFα, IFNγ, IL-6, IL-10 and IL-17 were tested using cytokine measuring kits (Invitrogen, USA) following the manufacturer's protocol. 50 µl of serum samples from 0-day, 7-day- and 14-day-infected mice were used to quantify the inflammatory response after post-operational (OP) observation due to infection.

### Immunogen preparation

OMVs were isolated from the *Helicobacter* strain [A61C (1), *cagA+, vacA s1m1*] following the methods described previously with slight modification (Bhaumik et al., 2023). In brief, BB broth (BD Biosciences, Difco, USA) was inoculated with log phase (OD_600_ ∼0.6) pre-culture of the respective strains and kept overnight in microaerophilic conditions under constant shaking (100 rpm) at 37°C. On the next day, centrifugation was performed consecutively first at 8000×***g*** for 15 min at 4°C, followed by 30 min with same conditions. The supernatants were then filtered twice with 0.45 μm and 0.22 μm syringe filters (Millipore, USA). To prevent protein degradation, a protease inhibitor cocktail was incorporated into the filtrate and ultracentrifuged at 140,000×***g*** at 4°C for 4 h using a P27A-1004 rotor (Hitachi). A density gradient centrifugation allowed obtaining the purified OMVs. Protein content was measured using a Lowry protein estimation kit (Pierce, USA) and stored at −20°C until further use.

### Characterization of the OMVs

#### Dynamic light scattering

Concentrated OMVs were diluted 10-fold to reach a concentration of 0.1 mg/ml. The hydrodynamic size of OMVs was measured using a Malvern Zetasizer ZS90 (Malvern Instruments, Germany) and analyzed using ZS Xplorer version 3.1.0.64 (Choi et al., 2017).

#### TEM

Diluted OMVs were placed on a carbon-coated grid and left for 10–20 min for absorption. The samples were then washed twice with drops of Tris buffer solution. Excess fluid was soaked using blotting paper, followed by staining with 2% uranyl acetate and air drying. Finally, the OMV-coated grids were observed under a JEOL JEM 2100 HR (JEOL, Tokyo, Japan) (Melo et al., 2021).

#### LC/MS of OMVs and analyses

Proteins present in OMVs were used for digestion and reduced with 5 mM TCEP and further alkylated with 50 mM iodoacetamide and again digested with trypsin (1:50, trypsin/lysate ratio) for 16 h at 37°C. Digests were cleaned using a C18 silica cartridge to remove the salt and dried using a speed vac. The dried pellet was resuspended in buffer A (2% acetonitrile, 0.1% formic acid). Experiments were performed on an Easy-nlc-1000 system coupled with an Orbitrap Exploris mass spectrometer. One microgram of peptide sample was loaded on a C18 column (15 cm, 3.0 μm Acclaim PepMap, Thermo Fisher Scientific), separated with a 0–40% gradient of buffer B (80% acetonitrile, 0.1% formic acid at a flow rate of 500nl/min) and injected for MS analysis. LC gradients were run for 60 min. MS1 spectra were acquired in the Orbitrap (MaxIT: 25 ms, AGQ target: 300%; RF lens: 70%; R: 60 K, mass range: 375–1500; profile data). Dynamic exclusion was employed for 30 s, excluding all charge states for a given precursor. MS2 spectra were collected for the top 12 peptides. MS2 (Max IT: 22 ms, R: 15 K, AGC target 200%). All samples were processed, and the generated RAW files were analyzed with Proteome Discoverer (version 2.5) against the UniProt organism database. For dual Sequest and Amanda searches, the precursor and fragment mass tolerances were set at 10 ppm and 0.02 Da, respectively. The protease used to generate peptides, i.e. enzyme specificity was set for trypsin/P (cleavage at the C-terminus of “K/R: unless followed by ‘P’”). Carbamidomethyl on cysteine as a fixed modification and oxidation of methionine and N-terminal acetylation were considered variable modifications for the database search.

### Extraction of LPS and OMPs

LPS and OMPs were extracted following the methods described earlier (Mukherjee et al., 2016). LPS was then treated with proteinase-K to ensure the absence of any protein residue. The carbohydrate content of LPS was then quantified using the phenol‒sulfuric acid method and measured at a wavelength of 492 nm (DuBois et al., 1956). Isolated proteins were quantified for their concentration using a Modified Lowry's Kit (Pierce, USA) and measured at 660 nm using a spectrophotometer.

### ELISA

Serum immunoglobulin (IgG, IgM, IgA, IgG2c) levels were measured against OMPs or LPS following the method described previously (Howlader et al., 2018). Twofold serial dilutions were prepared from serum isolated from both immunized and non-immunized groups. HRP-conjugated secondary anti-mouse IgG, anti-IgA, anti-IgG2c and anti-IgM antibodies (Sigma-Aldrich, USA) were used to detect the antibody titer. Each experiment was replicated thrice with pooled sera from different groups.

### Serum bactericidal assay (SBA) and SEM

The effect of immunized mouse sera on bacterial morphology was measured and visualized using SEM following a previously described protocol (Maiti et al., 2021). Bacteria along with heat-inactivated mouse sera and 25% guinea pig complement (with/without) were incubated for 1 h under microaerophilic conditions followed by plating for viable colonies or fixation with 3% glutaraldehyde overnight followed by a gradual dehydration step initially with alcohol and then substitution later with a mixture of alcohol and hexamethyldisilazane (HMDS) at ratios of 2:1, 1:1 and 1:2. Finally, the samples were mounted on specimen stubs, sputter-coated with gold and analyzed on a Quanta 200 SEM (FEI, the Netherlands).

### Cytokine assay

Both immunized and non-immunized mice were euthanized, and the spleens were harvested. After isolating spleen cells; ∼10^5^ cells were cultured for 2 h in RPMI1640 containing 10% FBS incubated with 50 μg of OMVs and incubated overnight at 37°C (with 5% CO_2_) for 24 h. IL-10, IFN-γ, IL-1β, IL-6, IL-4, TNF-α and IL-17 were measured in the culture supernatant using a cytokine measuring kit (Invitrogen, USA) (Ismail et al., 2003).

### FACS analysis

Spleen cells were harvested, cultured for 2 h in RPMI1640 containing 10% FBS and re-stimulated using isolated OMVs (50 μg) and incubated overnight at 37°C (with 5% CO_2_) for 24 h. The next day, the cells were scraped, washed thoroughly, blocked and then incubated with mouse anti-CD4+, CD8+ or CD19+ antibodies. Splenocytes were stained with anti-Mabs: CD4-phycoerithrin (PE), CD8 PE, CD19 PE or an isotype control PE (Miltenyi Biotec, USA). Unbound antibodies were washed, and a specific epitope of the immune cell population was observed using FACS Aria II (Maiti et al., 2021).

### Protective efficacy study

Seven days after the last immunization, both the immunized and non-immunized groups were challenged with the WT SS1 strain using a newly developed surgical procedure and housed for 7 days before being euthanized. The antrum of stomach of both immunized and non-immunized groups was isolated and separated into two parts. Half of each part was immediately transferred to BHI kept on ice, and the other half was transferred to neutral buffered formalin (NBF, 10%) solution to fix the tissue and left at room temperature. Harvested tissue in BHI was weighed, homogenized and serially diluted using PBS. The diluted samples were then spread onto BHIA and kept under microaerophilic conditions for 3–5 days. Any visible colonies were then counted and confirmed using RUT and PCR. Histopathological assays were performed as described elsewhere (Kim and Park, 2016; Hassan et al., 2016). Briefly, samples kept in 10% formalin were washed and gradually dehydrated using the alcohol gradation method followed by preparing a paraffin block. A thin section (approximately 5 μm) was prepared using a microtome. The slides were then de-waxed, rehydrated, and stained. H&E staining was used for the study because it enhances tissue or bacterial contrast. Finally, the slides were mounted and observed under a microscope. Histological scoring was assigned for each sample based on their morphological changes. The gastric tissue observed under a microscope revealed various degrees of gastritis, which was then categorized according to the Houston-updated Sydney system based on the infiltration of inflammatory cells within the lamina propria (Björkholm et al., 2002).

### Statistical analysis

The presented data do not follow a normal distribution due to biological variations. Nonparametric tests were adopted for all data analyses. Triplicate data were expressed as the mean±s.d. using GraphPad Prism version 5.02. Two-way ANOVA or the Mann‒Whitney test (for animal data) was performed as per the requirements, and statistical significance was determined from the *P*-values mentioned in the figure legends.

## Supplementary Material

10.1242/biolopen.060282_sup1 Supplementary information

Table S7.
