# Supplementary material for: Establishment of an intragastric surgical model using C57BL/6 mice to study the vaccine efficacy of OMV-based immunogens against Helicobacter pylori
Source: Biol Open. 2025 Jun 23;14(6):bio060282. doi: 10.1242/bio.060282 (PMC12233065; doi:10.1242/bio.060282)
Supplement: Supplementary information [file biolopen-14-060282-s1.pdf]

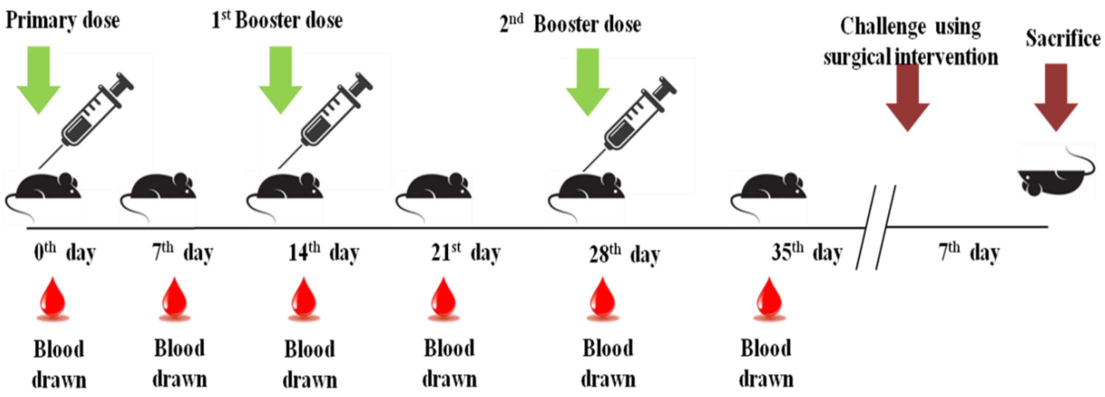

Fig. S1. Immunization and blood collection schedule Oral or Intraperitoneal (i.p.) route of immunization

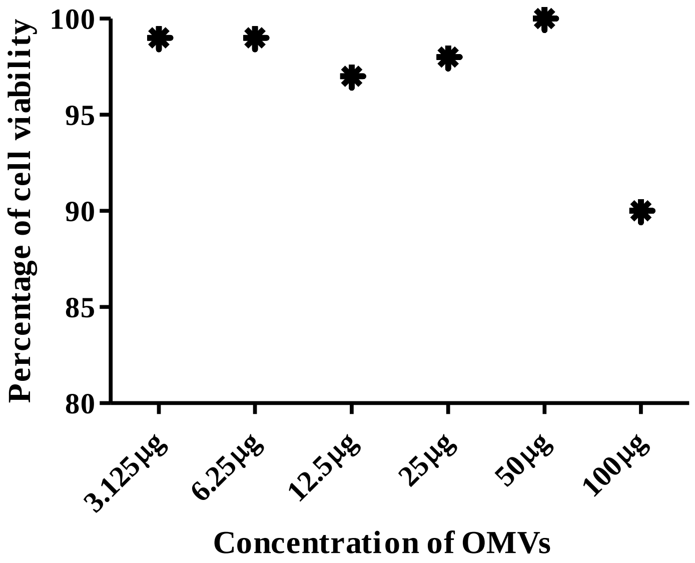

Fig. S2. *in-vitro* cytotoxicity test with different concentrations of OMVs

Biology Open • Supplementary information

Table S1. The Antibiotic profile of *Helicobacter pylori*used for immunogen strain selection.

Strains			Antibiotics					
			Clarithromycin		Amoxicillin		Metronidazole	
			S	R	S	R	S	R
1	Ref. strains	26695	+	-	+	-	-	+
		J99	+	-	+	-	-	+
		SS1	+	-	+	-	-	+
2	BHU 8A		-	+	+	-	-	+
3	KO 8A		+	-	+	-	-	+
4	AS 2		+	-	+	-	-	+
5	OT-10 (A)		+	-	+	-	-	+
6	B34		+	-	+	-	-	+
7	D383		+	-	+	-	-	+
8	B6		+	-	+	-	-	+
9	M28		+	-	+	-	-	+
10	L7		+	-	+	-	-	+
11	A61C(1)		+	-	+	-	-	+
12	AM1		+	-	+	-	-	+
S - sensitive					R - resistant			

Biology Open • Supplementary information

Table S2. Primers of specific genes of *Helicobacter pylori*used in this study.

Gene Assigned	Primer	Sequence	Amplicon (bp)	Reference
cagA	cag5c-F<br>cag3c-R	5′-GTTGATAACGCTGTCGCTTCA-3′<br>5′-GGGTTGTATGATATTTTCCATAA-3′	350	Chattopadhyay et al, 2004 [20]
vacA s1/s2	VA1-F<br>VAI-R	5′-ATGGAAATACAACAAACACAC-3′<br>5′-CTGCTTGAATGCGCCAAAC-3′	259/286	
vacA m1/ m2	VAG-F<br>VAG-R	5′-CAATCTGTCCAATCAAGCGAG-3′<br>5′-GCGTCAAAATAATTCCAAGG-3′	567/642	
babA2	babA2R<br>babA2F	5′-AATCCAAAAAGGAGAAAAAGTATGAAA-3′<br>5′-GTTTTCTTTGAGCGCGGGTAAGC-3′	607	Ghosh et al, 2016 [21]
ureB	ureBF<br>ureBR	5′-CGTCCGGCAATAGCTGCCATAGT-3′<br>5′-GTAGGTCCTGCTACTGAAGCCTTA-3′	480	Ghosh et al, 2016 [21]
dupA	jhp0917F<br>jhp0917R	5′-TGGTTTCTACTGACAGAGCGC-3′<br>5′-AACACGCTGACAGGACAATCTCCC-3′	307	Lu et al, 2005 [66]
	jhp0918F<br>jhp0918R	5′-CCTATATCGCTAACGCGCGCTC-3′<br>5′-AAGCTGAAGCGTTTGTAACG-3′	276	
16SrRNA	16SF<br>16SR	5′-CTGGAGAGACTAAGCCCTCC-3′<br>5′-ATTACTGACGCTGATTGCGC-3′	110	Kashyap et al., 2020 [67]

Table S3. Result of major virulence genes of *Helicobacter pylori*screened for immunogen strain selection

**Genetic Features**			**Virulence Marker**					**Adhesion**	**Duodenal Ulcer Promoting Gene**	
**Strains**			***cagA***	***vacA***				***babA2***	***dupA***	
				s1	***s2***	***m1***	***m2***		***jhp0917***	***jhp0918***
**1**	**Ref. strains**	**26695**	+	+	-	+	-	-	-	-
		**J99**	+	+	-	+	-	+	+	+
		**SS1**	+	-	+	-	+	-	-	-
**2**	**BHU 8A**		+	+	-	-	+	+	-	-
**3**	**KO 8A**		+	+	-	+	-	+	-	-
**4**	**AS 2**		+	+	-	+	-	-	-	-
**5**	**OT-10 (A)**		+	+	-	+	-	-	-	-
**6**	**B34**		+	+	-	+	-	+	-	-
**7**	**D383**		-	-	+	-	+	-	-	-
**8**	**B6**		+	+	-	-	+	+	-	-
**9**	**M28**		+	+	-	-	+	-	-	-
**10**	**L7**		+	+	-	+	-	+	-	-
**11**	**A61C(1)**		+	+	-	+	-	+	+	+
**12**	**AM1**		-	-	+	-	+	-	-	-

Biology Open • Supplementary information

Table S4. Proteomic analyses of OMVs isolated from*Helicobacter pylori*strain A61C(1).

Accession	Protein assigned	Molecular mass (kDa)	No. of peptides	Sequence coverage (%)
P69996	nickel cation binding, urease activity	61.6	3	7
P14916	nickel cation binding, urease activity	26.5	1	4
P52093	ferric iron binding, ferrous iron binding, ferroxidase activity	19.3	2	19
P42383	ATP binding, ATP-dependent protein folding chaperone, isomerase activity, unfolded protein binding	58.2	3	5
O25723	transmembrane transporter activity	47.5	1	9
O26011	carboxy-lyase activity, flavin prenyltransferase activity	20.6	1	18
P56003	GTP binding, GTPase activity, guanosine tetraphosphate binding, translation elongation factor activity	43.6	2	4
O25475	ABC-type protein transporter activity, ATP binding, metal ion binding, protein-exporting ATPase	99	1	3

Biology Open • Supplementary information

	activity			
O24949	Hypothetical protein	23.6	1	3
O26012	Hypothetical protein	24.3	1	3
O25206	ATP binding, DNA binding	98.4	1	3
P56035	rRNA binding, structural constituent of ribosome	16.5	1	9
O25095	ATP binding, tetraacyldisaccharide 4'-kinase activity	35.5	1	5
O25434	4 iron, 4 sulfur cluster binding, aspartic acid methylthiotransferase activity, metal ion binding,proteinmethylthiotransferase activity	49.6	1	8
O26001	ATP binding,ATP hydrolysis activity	29.2	1	14
O25992	Hypothetical protein	82.3	1	3
P56081	3-dehydroquinate synthase activity, metal ion binding, nucleotide binding	39.1	1	3

Biology Open • Supplementary information

Table S5. Sydney classification of histopathological scoring used in the study

	Neutrophil infiltration	Glandular atrophy	Intestinal metaplasia	Chronic inflammation
**Grade I**	<1/3 of surface infiltrated	Mild	<1/3 of surface infiltrated	5-10 cells x 40
**Grade II**	1/3- 2/3 of surface infiltrated	Moderate	1/3- 2/3 of surface infiltrated	11-20 cells x 40
**Grade III**	>2/3 of surface infiltrated	Severe	>2/3 of surface involved	>21cells x 40

Scores assigned: mild=1, moderate=2, severe=3

Table S6. List of Antibodies used in the study

Sl no.	Antibody	Catalog no	Company
1.	Anti mouse-IgA	AB97235	Abcam
2.	Anti mouse-IgG2c	AB97255	Abcam
3.	Anti mouse-IgG	AB97023	Abcam
4.	Anti mouse-IgM	A8786	Sigma aldrich

Table S7.

Available for download at
https://journals.biologists.com/bio/article-lookup/doi/10.1242/bio.060282#supplementary-data
